# A key centriole assembly interaction interface between human PLK4 and STIL appears to not be conserved in flies

**DOI:** 10.1242/bio.024661

**Published:** 2017-02-15

**Authors:** Matthew A. Cottee, Steven Johnson, Jordan W. Raff, Susan M. Lea

**Affiliations:** Sir William Dunn School of Pathology, University of Oxford, Oxford OX1 3RE, UK

**Keywords:** Centriole duplication, Centrosome, Cartwheel

## Abstract

A small number of proteins form a conserved pathway of centriole duplication. In humans and flies, the binding of PLK4/Sak to STIL/Ana2 initiates daughter centriole assembly. In humans, this interaction is mediated by an interaction between the Polo-Box-3 (PB3) domain of PLK4 and the coiled-coil domain of STIL (HsCCD). We showed previously that the *Drosophila* Ana2 coiled-coil domain (DmCCD) is essential for centriole assembly, but it forms a tight parallel tetramer *in vitro* that likely precludes an interaction with PB3. Here, we show that the isolated HsCCD and HsPB3 domains form a mixture of homo-multimers *in vitro*, but these readily dissociate when mixed to form the previously described 1:1 HsCCD:HsPB3 complex. In contrast, although *Drosophila* PB3 (DmPB3) adopts a canonical polo-box fold, it does not detectably interact with DmCCD *in vitro*. Thus, surprisingly, a key centriole assembly interaction interface appears to differ between humans and flies.

## Abstract (precis)

**Summary:** PLK4 and STIL/Ana2 proteins interact to promote centriole duplication. We show that these proteins may homo-multimerise in multiple ways, and that their interaction is likely complex and may differ between species.

## INTRODUCTION

Centrioles form centrosomes and cilia, two organelles that have many important functions (Bettencourt-Dias et al., 2011; Conduit et al., 2015). Centriole duplication is tightly regulated and recent studies suggest that only a small number of conserved proteins are essential for this process (Conduit et al., 2015; Jana et al., 2014). Centriole assembly is initiated when CEP192/Spd-2 and/or CEP152/Asl recruit the protein kinase PLK4/Sak to the mother centriole (Kim et al., 2013; Park et al., 2014; Pelletier et al., 2006; Sonnen et al., 2013). PLK4/Sak then recruits STIL/Ana2, activating the kinase and allowing it to phosphorylate STIL/Ana2, which can then interact with and recruit Sas-6 (Dzhindzhev et al., 2014; Kratz et al., 2015; Moyer et al., 2015; Ohta et al., 2014). Sas-6 and STIL/Ana2 cooperate to initiate the assembly of the central cartwheel (Stevens et al., 2010b), and STIL/Ana2 directly recruits Sas-4 (Cottee et al., 2013; Hatzopoulos et al., 2013; Tang et al., 2011), which helps recruit MTs around the cartwheel (Hsu et al., 2008; Pelletier et al., 2006).

Although these core centriole duplication proteins often exhibit low levels of amino-acid homology between species, several interaction interfaces have now been structurally characterised and, so far, these interfaces are very similar (Cottee et al., 2013, 2015; Hatzopoulos et al., 2013; Kitagawa et al., 2011; Park et al., 2014; Shimanovskaya et al., 2014; van Breugel et al., 2011, 2014). Thus, unsurprisingly, it seems that the molecular interactions required for centriole assembly are well conserved between species.

**Fig. 1.**
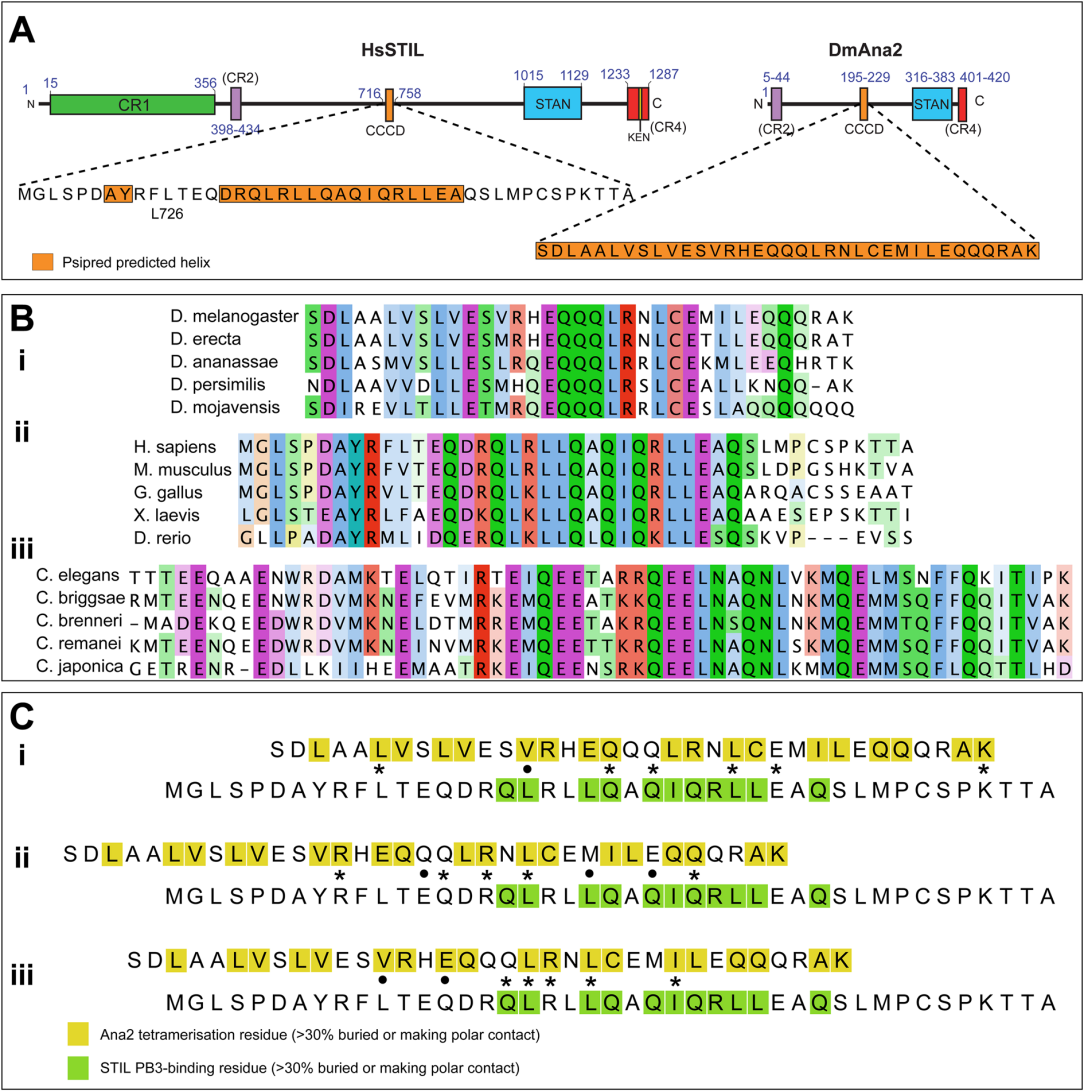
**Sequence analysis of CCD domains of STIL/Ana2 family proteins.** (A) Schematic illustration of the domain topologies of *Homo* *sapiens* STIL and *Drosophila* *melanogaster* Ana2. The sequences of the CCD domains are shown; regions predicted to be helical (Jones, 1999) are highlighted in orange. (B) Multiple sequence alignments of CCD regions from (i) *Drosophila*, (ii) vertebrates, and (iii) *Caenorhabditis*, coloured according to the ClustalX scheme. Sequences within each phylum/genus align unambiguously, but alignments between these groups are poor and are ambiguous. (C) When STIL/Ana2 family proteins are included in multiple sequence alignments, the CCD regions often align, but the exact register of these alignments is often different. i, ii, iii show alignments of the HsSTIL and DmAna2 CCD domain sequences extracted from different multiple sequence alignments. Asterisks represent identical residues and dots indicate similar residues. Residues known to be involved in either tetramerisation (Ana2) or in PB3 binding (STIL) are coloured yellow or green, respectively. The structures of the Ana2 CCD tetramer (PDB ID: 5AL6) and the STIL CCD in complex with PLK4-PB3 (PDB ID: 4YYP) were analysed by the PISA server (Krissinel and Henrick, 2007). In each alignment, CCD residues involved in the relevant interfaces are coloured according to the legend. Each alignment is unique, but in each case a similar number of residues appear conserved. No single alignment appears more feasible than any other, so it is not possible to unambiguously align these sequences.

STIL/Ana2 proteins generally contain several conserved regions (Fig. 1A) including a STAN domain (Stevens et al., 2010a) implicated in binding Sas-6 (Dzhindzhev et al., 2014; Ohta et al., 2014), a short N-terminal region (CR2) that binds Sas-4 (Cottee et al., 2013; Hatzopoulos et al., 2013), and a predicted coiled-coil domain (CCD) usually located close to the centre of the protein (Goshima et al., 2007; Stevens et al., 2010a). Vertebrate STIL proteins also have an extended N-terminal conserved region (CR1) that appears to be vertebrate specific. The CCD seems to be essential for function in all species. *Drosophila* Ana2-CCD (DmCCD) is required to localise Ana2 to centrioles and it forms a tight parallel tetramer; mutations that perturb tetramer assembly *in vitro* strongly perturb centriole assembly *in vivo*, suggesting that Ana2 homo-oligomerisation is functionally important (Cottee et al., 2015). In *Caenorhabditis* *elegans* SAS-5 is the functional homologue of Ana2, and the SAS-5-CCD also multimerises and is essential for function; although the SAS-5-CCD forms a trimer *in vitro*, the SAS-5 protein can assemble into higher-order multimers through an additional multimerisation domain (Dynes et al., 2015). The human STIL-CCD (HsCCD) also multimerises *in vitro* (Cottee et al., 2015) and appears to be essential for function (Arquint et al., 2015; David et al., 2016). The HsCCD is required for STIL self-association *in vivo*, but an HsCCD monomer also forms an antiparallel coiled-coil interaction with a monomeric PB3 domain of PLK4, and this interaction targets STIL to centrioles (Arquint et al., 2015).

Thus, in all STIL/Ana2/SAS-5 molecules studied to date, the CCD plays a vital role in centriole assembly, but it is unclear whether this is because it allows homo-multimerisation, the interaction with the PB3 domain of PLK4, or both. Furthermore, conflicting structural information has also been reported for the PLK4 PB3 domain of humans and mice, with the human PB3 domain behaving as a monomer (Arquint et al., 2015) and the mouse PB3 domain behaving as an unusual strand-swapped dimer (Leung et al., 2002). It is unclear whether this reflects genuine species differences. This point is potentially important, as the ability of PLK4 to multimerise and autophosphorylate *in trans* is crucial to its regulation (Cunha-Ferreira et al., 2013; Guderian et al., 2010; Holland et al., 2010). Here, we attempt to resolve some of these issues by studying the structures and interactions of CCDs and PLK4-PB3s in humans and flies.

## RESULTS AND DISCUSSION

In our previous study we demonstrated that DmCCD is tetrameric *in crystallo* and *in vitro* under all conditions tested, while HsCCD formed concentration-dependent multimers *in vitro* (Cottee et al., 2015). To further characterise this difference, we sought to solve the structure of the HsCCD. Although the predicted CCD regions are well conserved within vertebrate, fly and worm species (Fig. 1B), they are poorly conserved between these groups and it is difficult to unambiguously align the sequences of the human STIL-CCD with the worm or fly CCDs (see, for example, Fig. 1C). This ambiguity in alignment means it is not possible to predict whether, and if so how, the DmCCD and DmPB3 domains might interact.

**Fig. 2.**
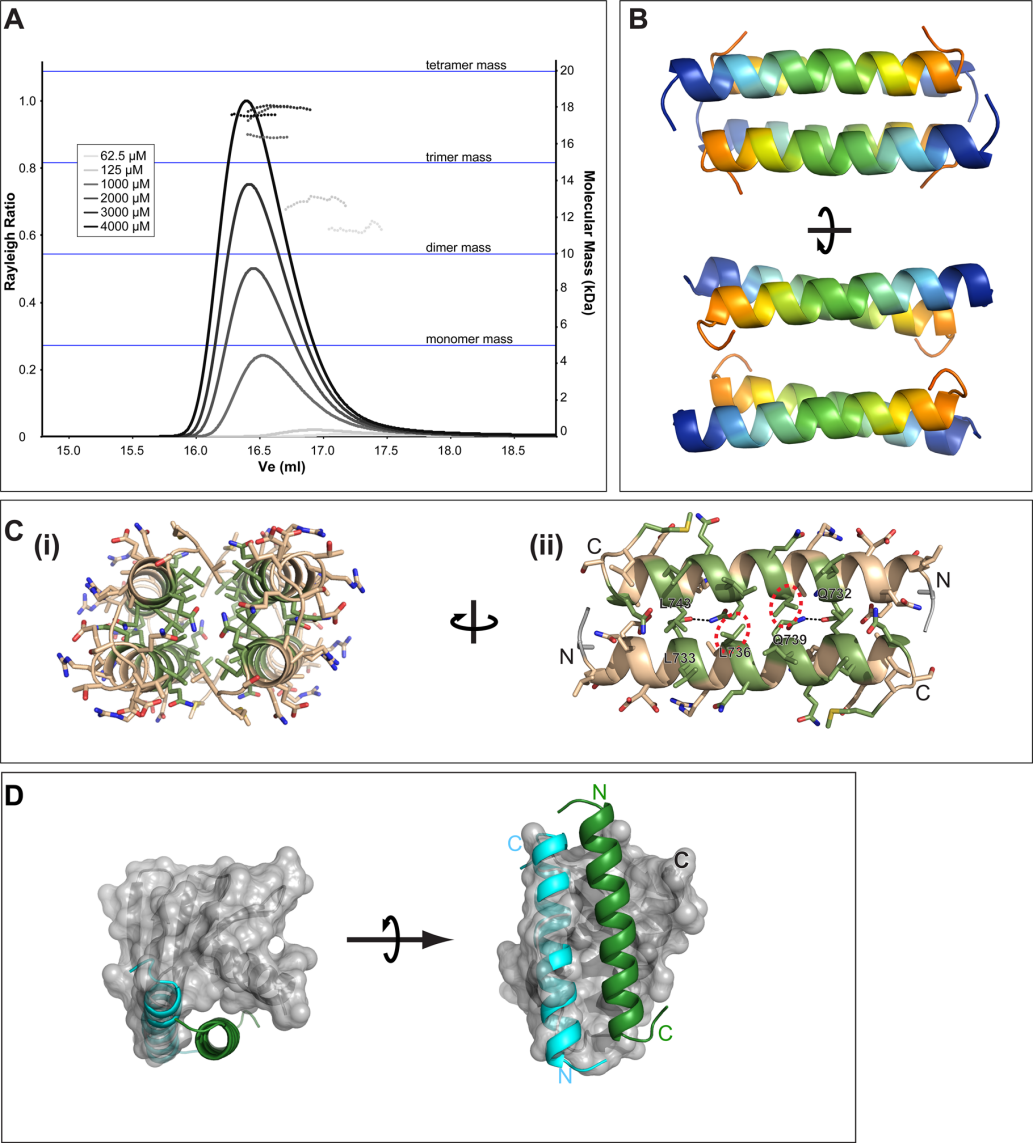
**The STIL CCD forms unstable oligomers in solution and crystallises as an antiparallel dimer of dimers.** (A) SEC-MALS analysis of the STIL CCD (aa 717-758). This construct differs slightly from that used in our previous study (Cottee et al., 2015) (see Materials and Methods). Different injected protein concentrations are indicated by different shades of grey, as indicated. Solid lines represent the relative Rayleigh ratio and dashed lines show the measured masses across each peak. For reference, horizontal blue lines indicate the masses of a monomer, dimer, trimer and tetramer. The STIL CCD can be seen to self-associate in solution. The average mass of these assemblies increases with concentration and varies between dimeric to nearly tetrameric. 100 µl of each sample was injected over an S200 10/300 column. (B) The crystal structure of the STIL CCD (aa 726-750) at 0.91 Å reveals a symmetric, anti-parallel coiled-coil dimer of dimers generated by crystallographic symmetry. Each helix is shown as a cartoon coloured blue→orange, N→C. (C) (i) End-on view of the CCD anti-parallel dimer of dimers, shown as a tan cartoon and stick representation; residues that form the CCD:PB3 interface are coloured in green. (ii) Expanded view of the most closely associated dimer. Highlighted by a dashed red circle is residue L736, which is involved in both the dimerisation and PB3 interfaces. Mutation of this residue affects both STIL self-oligomerisation and PB3 binding (David et al., 2016). (D) Superposition of the dimer of HsCCD onto the previously published HsPB3:HsCCD structure (4YYP). The first HsCCD helix is modelled as a green cartoon in the HsPB3 binding site. The second copy of the STIL-CCD helix is shown as a blue cartoon and clashes with several PB3 loops (grey surface), indicating that HsCCD self-association and binding to PB3 are likely mutually exclusive.

We combined secondary structure predictions and coiled-coil analysis to design multiple constructs in the CCD region of STIL. In agreement with our previous study using an HsCCD peptide, size exclusion chromatography – multi-angle laser light scattering (SEC-MALS) analysis of purified HsCCD revealed that it showed concentration-dependent oligomerisation. Although the average mass never fell below that of a dimer at lower concentrations (62 µM), it never quite reached that of a tetramer at higher concentrations (4000 µM) (Fig. 2A) (Cottee et al., 2015). We solved the crystal structure of HsCCD to 0.91 Å (Table 1), revealing that, in contrast to the parallel coiled-coil tetramer formed by DmCCD, HsCCD formed an anti-parallel coiled-coil tetramer in the crystal (Fig. 2B). Consistent with the solution data, two helices packed in an anti-parallel arrangement to form a tight coiled-coil dimer, with the tetramer being formed from a less tight association of two dimers. Interestingly, many amino acids previously demonstrated to be involved in PB3-binding are buried in the dimer and tetramer interface (amino acids highlighted in green, Fig. 2C). Furthermore, superposition of the more tightly associated dimer onto the existing crystal structure of a HsCCD/HsPB3 complex (PDB ID: 4YYP) demonstrated that the second monomer of the HsCCD dimer would sterically clash with PB3 (Fig. 2D), and so PB3 would not be able to bind HsCCD in the tight dimeric form we observe in our structure. This strongly suggests that HsCCD self-association and binding to PB3 are mutually exclusive events.

**Table 1.**
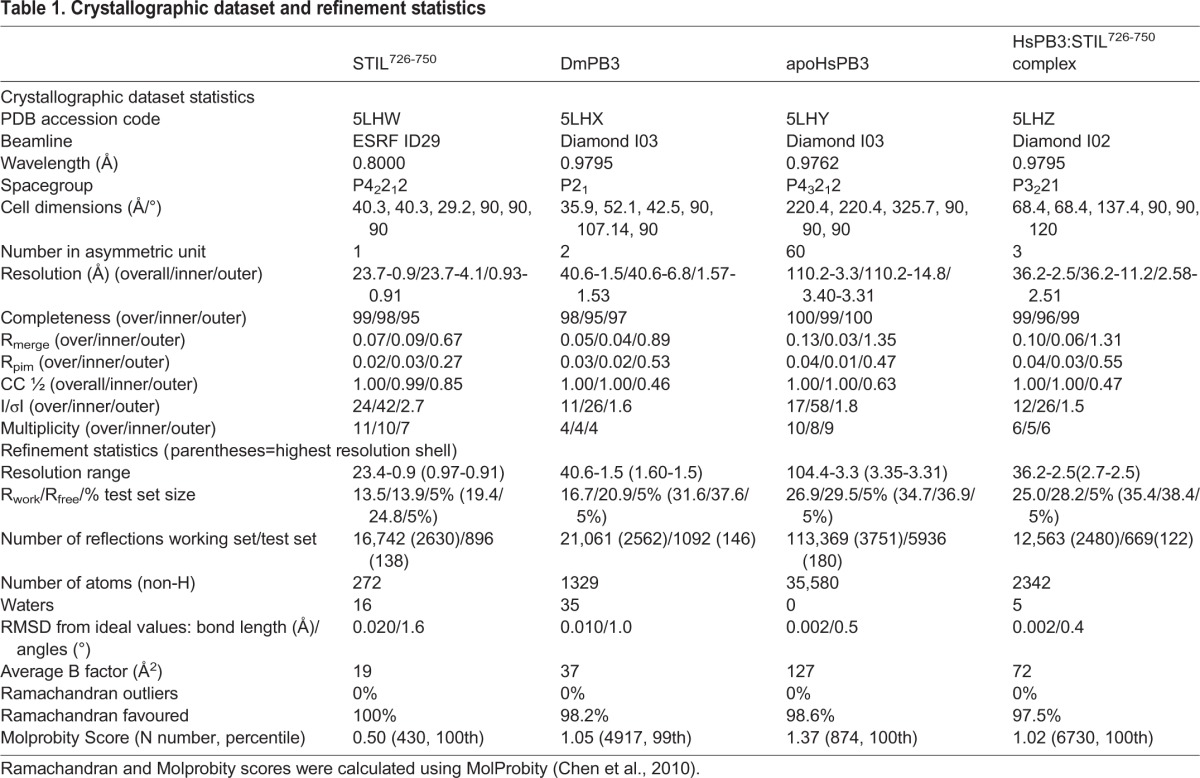
**Crystallographic dataset and refinement statistics**

**Fig. 3.**
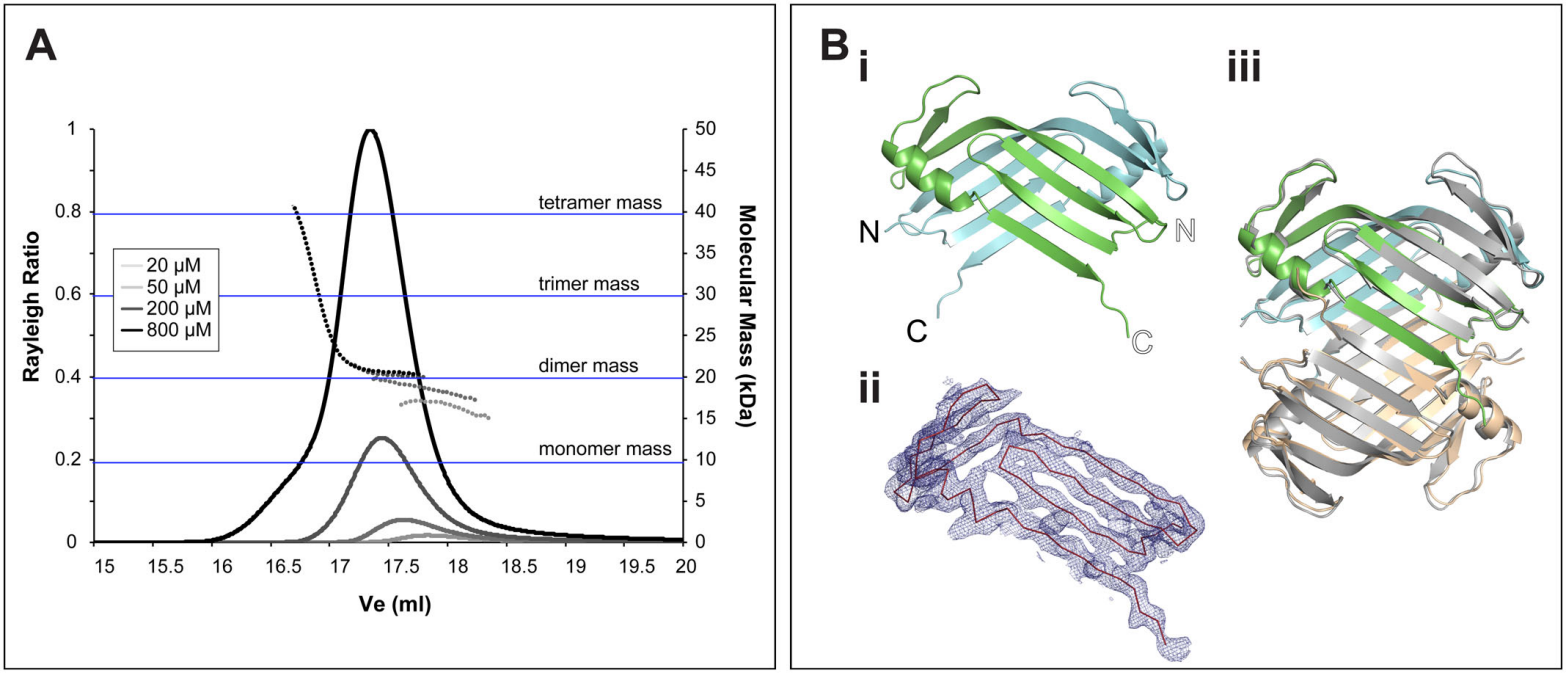
**apoHsPB3 forms a strand-swapped dimer of dimers.** (A) SEC-MALS analysis of apoHsPB3 (aa884-970). Apo HsPB3 eluted as a single peak. Solid lines represent the relative Rayleigh ratio and dashed lines show the measured masses across each peak. 100 µl of apo HsPB3 at 200 µM was injected over a Superdex 200 10/300 column. (B) (i) Apo HsPB3 crystallised as a strand-swapped dimer. 60 chains were present in the asymmetric unit (ASU) of the crystal, forming 30 virtually identical strand-swapped dimers, exhibiting very strong non-crystallographic symmetry. Chains Y (green) and Z (blue) are shown in cartoon representation. (ii) The tertiary structure of the strand swapped dimers is unambiguous. Electron density map (blue mesh) carved around apo HsPB3 chain Y, at 1.3σ, showing contiguous density throughout the backbone of the chain. apo HsPB3 chain Y is shown in red ribbon representation. (iii) The HsPB3 strand-swapped dimer (green, blue and tan cartoons) forms a tetramer that is very similar to that seen in the mouse PB3 crystal (MmPB3, grey cartoon).

In light of these results, we sought to confirm that we could reproduce the previously identified interaction between HsCCD and HsPB3 (Arquint et al., 2015). In this previous study, HsPB3 behaved as a monomer in solution, and its structure was solved by nuclear magnetic resonance spectroscopy (NMR). In our hands, however, HsPB3 was seen to self-associate in solution, forming oligomers with masses up to that of a tetramer (Fig. 3A). We solved the crystal structure of HsPB3 to 3.3 Å (Table 1), revealing that it formed a strand-swapped dimer (Fig. 3Bi, Bii) that further assembled into a tetramer (green, blue and tan chains, Fig. 3Bii), consistent with the SEC-MALS data. Further analysis of the crystal packing revealed that the strand-swapped dimer was equivalent to that reported previously for mouse PB3 (mPB3) (Leung et al., 2002) (RMSD=0.7 Å over 140 Cα atoms) and that, surprisingly, a nearly identical tetrameric assembly was also observed in the mPB3 crystals (grey chains, Fig. 3Biii) (RMSD=1.2 Å over 300 Cα atoms). Crucially, the spacegroup and packing arrangement of the HsPB3 and the MmPB3 crystals were unrelated, indicating that this unusual strand-swapped tetramer is unlikely to simply be a crystallization artefact, although we cannot exclude this possibility entirely.

**Fig. 4.**
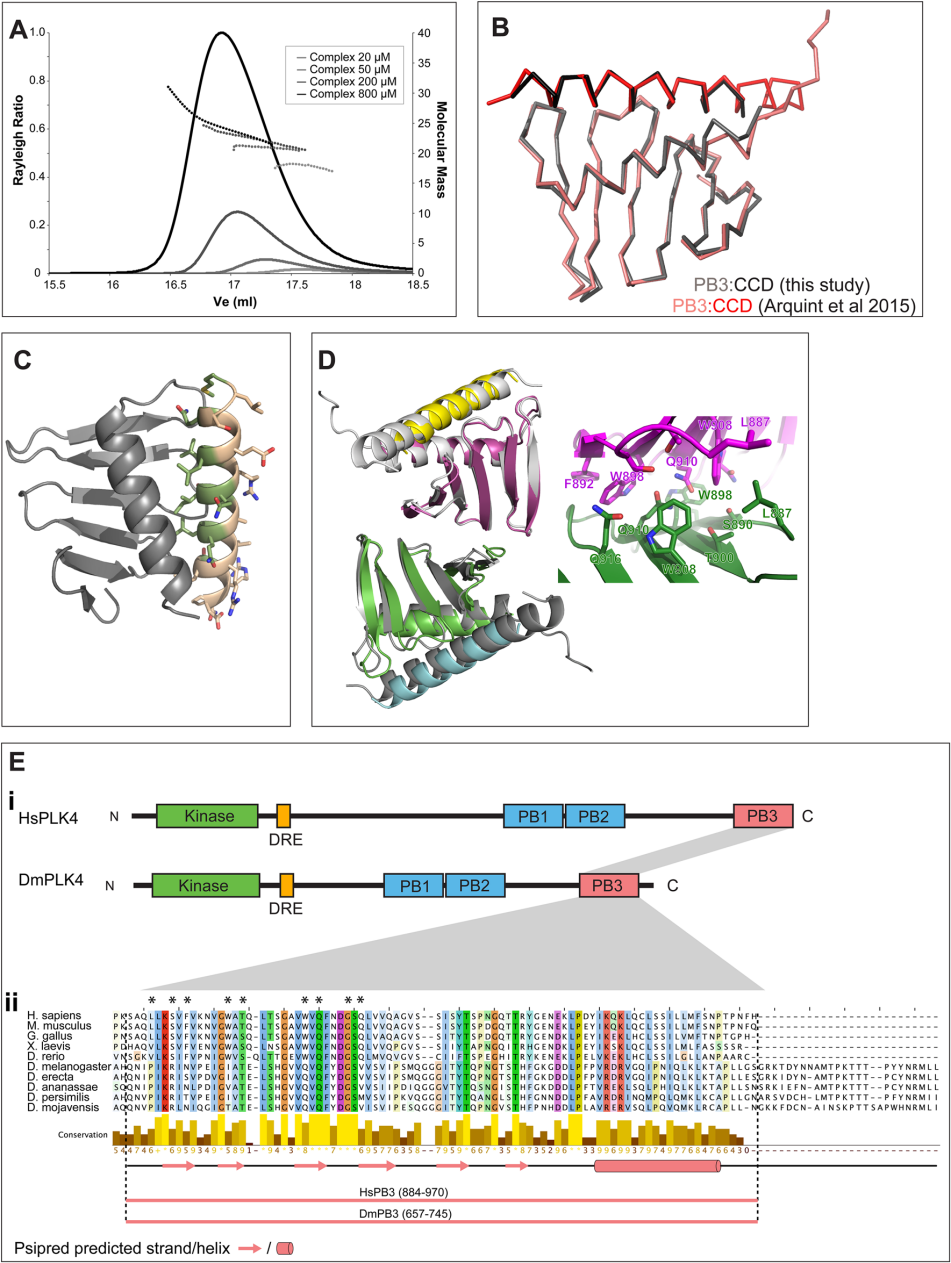
**HsPB3 and HsCCD form a complex.** (A) SEC-MALS analysis of HsPB3 mixed with HsCCD at various concentrations. Solid lines represent the relative Rayleigh ratio and dashed lines show the measured masses across each peak. 100 µl of each sample was injected over an S200 10/300 column. (B) Ribbon overlay of the PB3:CCD complex (grey:black, this study) with that previously reported (Arquint et al., 2015) (pink:red). The complexes overlay with a root-mean-square deviation (RMSD) of 0.535±0.053 Å over 85±4 Cα atoms. (C) The complex of HsPB3 (grey) with the STIL CCD (tan) in cartoon representation (this study). Three such copies were evident in the crystal ASU. CCD residues interfacing with PB3 are coloured green. (D) Overlay of a dimer of heterodimers from the HsPB3:STIL-CCD crystal (coloured cartoon) with an equivalent assembly observed in the earlier structure 4YYP (grey cartoon). Inset is a zoom on the dimer interface highlighting the highly hydrophobic nature of the interaction. (E) (i) Schematic illustration showing the domain topologies of the PLK4 orthologues from humans and *D. melanogaster*. (ii) Multiple sequence alignment of the PLK4 PB3 domain sequences from five vertebrates and five *Drosophila* species. The sequences align well and are predicted (Jones, 1999) to share similar secondary structures as annotated below the alignment. Shown below this are the domain boundaries of the HsPB3 and DmPB3 constructs used in this study. These boundaries were chosen to be topologically equivalent to other PB3 constructs used in previous studies (Arquint et al., 2015; Leung et al., 2002). Residues involved in the HsPB3:HsPB3 interaction interface shown in D are highlighted with asterisks.

As the multimers observed for both HsPB3 and HsCCD were in conflict with the previously observed 1:1 complex formed between these proteins, we set out to reanalyse their interaction. SEC-MALS analysis of a mixture of the two components indicated a complex range of oligomeric species that showed concentration dependence (Fig. 4A). We therefore solved the crystal structure of the complex to 2.5 Å (Table 1), confirming the structure of the previously reported 1:1 dimer (average RMSD=0.5 Å) (Fig. 4B,C) (Arquint et al., 2015). Intriguingly, however, our crystal contained multiple copies of the 1:1 complex in the asymmetric unit and packed to form dimers of the heterodimer, i.e. a 2:2 complex (coloured chains, Fig. 4D). Strikingly, a nearly identical 2:2 complex (RMSD=0.9 Å over 196 Cα atoms) can also been seen in the earlier crystal form (4YYP) (Arquint et al., 2015), where the dimer is formed by one of the crystallographic twofold axes (grey chains, Fig. 4D). The interface is conserved between these two crystal forms despite the other crystal packing interfaces being completely different. This new interface is formed via the β-sheet of the PB3 domain and involves hydrophobic residues on the opposite face to the HsCCD binding site; interestingly, these residues are highly conserved from human to zebrafish, but are mostly not conserved when compared with the *Drosophila* PB3 (residues highlighted with an asterisk, Fig. 4Eii).

**Fig. 5.**
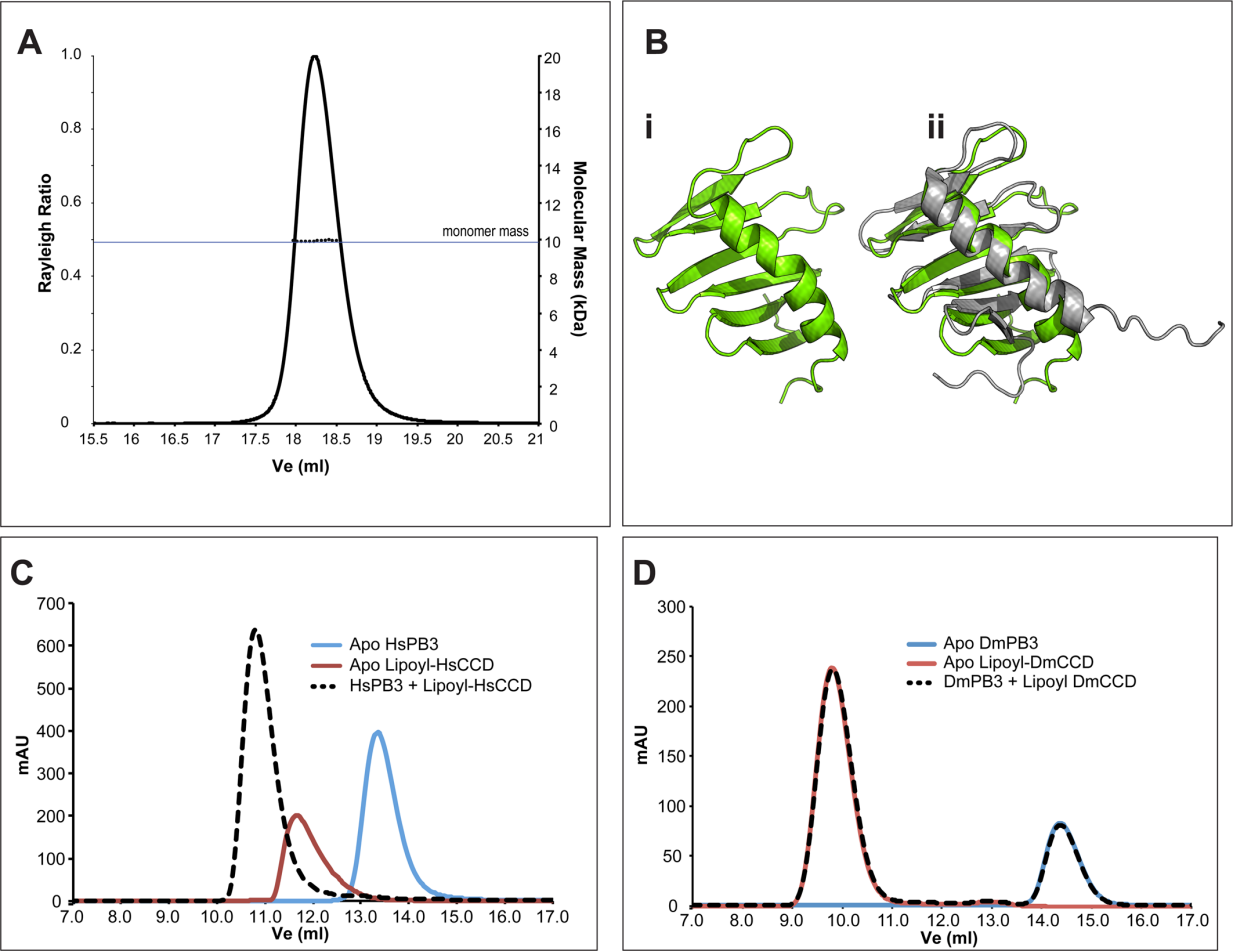
**Drosophila PB3 and CCD do not detectably interact.** (A) SEC-MALS analysis of DmPB3. Solid lines represent the relative Rayleigh ratio and dashed lines show the measured masses across each peak. 100 µl of DmPB3 at 500 µM was injected over an S200 10/300 column. (B) (i) The crystal structure of DmPB3 at 1.53 Å shown in green in cartoon representation. The domain exhibits a canonical Polo domain fold, forming a sequential six-stranded beta sheet with an alpha helix packed against one side. (ii) As in i, but with the NMR structure of HsPB3 (Arquint et al., 2015) (PDB ID: 2N19) superimposed as a grey cartoon (RMSD=1.3 Å over 58 Cα atoms). (C) Overlaid chromatograms of analytical gel filtration (AGF) experiments on the apo HsPB3 (blue trace) and apo lipoyl-HsCCD (red trace) domains. When the two proteins were mixed, the apo peaks were no longer evident, and a larger peak (dashed black trace) was evident indicating the formation of a complex. All proteins were injected at 500 µM in each experiment. (D) Overlaid chromatograms showing the equivalent experiment to C but carried out with apo DmPB3 (blue trace) and apo lipoyl-DmCCD (red trace). When these proteins were mixed (dashed black trace), the behaviour of the apo-proteins was not detectably altered.

We next wanted to test whether fly DmPB3 and DmCCD could form a complex similar to that formed by HsPB3 and HsCCD. Although HsPB3 and DmPB3 are generally well conserved (Fig. 4E), DmPB3 behaved as a monomer in solution (Fig. 5A). We solved the crystal structure of DmPB3 to 1.5 Å (Table 1), revealing that the DmPB3 monomer (green chain, Fig. 5Bi, Bii) adopted a typical Polo-Box fold that was very similar in structure to the monomeric HsPB3 (PDB ID: 2N19) previously solved from NMR studies (grey chain, Fig. 5Bii) (Arquint et al., 2015) (RMSD=1.3 Å over 58 Cα atoms). Interestingly, in contrast to the situation with HsPB3 and HsCCD (Fig. 5C), when we mixed the DmPB3 monomer and the DmCCD tetramer we could not detect any interaction (Fig. 5D), suggesting a lack of direct equivalence between the human and *Drosophila* systems.

Our results have several important implications for our understanding of the centriole assembly pathway. It is widely accepted that an interaction between PLK4/Sak and STIL/Ana2 plays an essential part in centriole assembly (Arquint et al., 2015; Dzhindzhev et al., 2014; Kratz et al., 2015; Moyer et al., 2015; Ohta et al., 2014). Surprisingly, our results indicate that the two proteins may physically interact in different ways in different species. In humans, the STIL-CCD forms a coiled-coil interaction with PLK4-PB3 that is required for centriole duplication, but our data suggests that the equivalent fly proteins do not interact in this way. This may explain why the CCD is well conserved within the vertebrates, insects and worms, but is not well conserved between these groups (Fig. 1B). Interestingly, the HsCCD can also interact with an additional linker region (L1) of PLK4 (Arquint et al., 2015); perhaps this interaction interface is conserved in flies and allows fly Sak/PLK4 and Ana2 to interact in the absence of the PB3:CCD interaction. Moreover, the HsCCD can also interact with Cdk1 (Zitouni et al., 2016), suggesting that the STIL-CCD may act as a platform for several different protein-protein interactions (self-oligomerisation, PLK4-PB3, PLK4-L1, Cdk1); many of these are likely to be mutually exclusive events due to the limited size of the CCD.

The PB3 domain of PLK4/Sak proteins is highly conserved and can target PLK4/Sak to centrioles (Leung et al., 2002). Our data, combined with previous studies, suggest that, when expressed in isolation, this domain can adopt several conformations: a monomer that exhibits a classical PB fold – as exhibited in the crystal structure of fly PB3 (this study) and the NMR structure of human PB3 (Arquint et al., 2015) – and an unusual strand-swapped multimer (either a dimer or tetramer) – as exhibited in the crystal structures of the human (this study) and mouse PB3 (Leung et al., 2002). In this study we observe that the HsPB3 construct can adopt both conformations: in its apo form, the HsPB3 shows a concentration­-dependent equilibrium between a strand-swapped multimer and a monomer, with the multimeric forms dominating. After the addition of HsCCD the HsPB3 crystallised as a canonical PB domain, in a 1:1 complex with STIL-CCD, indicating the PB3 strand-swapped multimer must have undergone a dramatic remodelling. The significance of this dual conformation of PB3 is unclear, although such plastic segment swapping has been linked to multi-domain protein evolution (Szilágyi et al., 2012) and amyloidogenesis (Wahlbom et al., 2007). The ability of PLK4 to multimerise is, however, crucial for regulating PLK4 stability (Cunha-Ferreira et al., 2009, 2013; Holland et al., 2010, 2012; Klebba et al., 2013; Rogers et al., 2009). Thus, regulated dimerisation/multimerisation through the PB3 domain, in conjunction with the characterised dimerisation of the cryptic Polo-box region of PLK4 (Park et al., 2014; Shimanovskaya et al., 2014; Slevin et al., 2012), could potentially play a part in regulating PLK4 activity.

Finally, although the STIL/Ana2 CCD is essential for centriole assembly, our results suggest that its function may differ between species. We speculated that the very tight parallel tetramer formed by the DmCCD might stabilise interactions that help ensure the invariant ninefold symmetry of the cartwheel (Cottee et al., 2015). This model remains plausible in flies, but appears unlikely in humans, as the HsCCD forms an antiparallel multimer that can readily dissociate to interact with PB3. Perhaps the simplest explanation for these findings is that although PLK4/Sak and STIL/Ana2 proteins can interact with themselves and with each other in different ways in different species, the sum of these interactions (and their interactions with other key centriole assembly proteins such as Sas-6 and Sas-4/CPAP) allows them to fulfil conserved functions in all species – even if the precise molecular interactions differ between species. All STIL/Ana2 proteins could, for example, ultimately be bound in the cartwheel in a similar conformation but, in flies, this conformation may be primarily dictated by the DmCCD, whereas in humans it might be dictated by other interactions. Alternatively, it may be that the interaction between PLK4/Sak and STIL/Ana2 is similar in flies and humans but is regulated in flies, perhaps by post-translational regulation.

## MATERIALS AND METHODS

### Protein expression constructs

DNA sequences encoding the CCD region of Ana2 (193-229) and STIL (717-758 or 726-750) were cloned into a custom ‘pLip’ vector similar to that described previously (Cottee et al., 2013, 2015), which encodes two TEV-cleavable His-tagged lipoyl domains from *Bacillus stearothermophilus* dihydrolipoamide acetyltransferase that flank the insert. In this study, two TAA stop codons were added after the CCD sequence in order to avoid the C-terminal EFGENLYFQ cleavage remnant. As a result, the expressed fusion protein contains only a single His-lipoyl tag, at the N-terminus of the CCD. Cleavage of this tag results in only a GGS remnant at the N-terminus of the CCD. DNA encoding the *Drosophila* Sak/PLK4 PB3 domain (657-745) or the human PLK4 PB3 domain (884-970) was PCR-amplified from a Sak/PLK4 cDNA clone or an *Escherichia* *coli* codon-optimised cDNA (Geneart), respectively. PB3 inserts were cloned into a pETM-44 vector (EMBL) encoding a 3C-cleavable N-terminal His-MBP tag, leaving a ‘GPMG’ cleavage remnant at the N-terminus of the PB3 constructs.

### Protein expression and purification

CCD fragments were expressed in *E. coli* C41 cells in LB broth and purified using Ni-NTA affinity chromatography. Lipoyl-CCD fusion constructs used for analytical gel filtration experiments were at this stage purified by size-exclusion chromatography. For MALS and crystallography, CCD fragments were cleaved and purified from their His-lipoyl tags using TEV protease followed by reverse Ni-NTA affinity and size-exclusion chromatography. Human and fly MBP-PB3 domains were expressed in *E. coli* B834 cells in LB broth and proteins were purified using Ni-NTA affinity, proteolytic (3C) cleavage, reverse Ni-NTA affinity and size exclusion. DmPB3 eluted in a single monomeric peak (by MALS analysis), while HsPB3 eluted as a single dimeric peak. To prepare the HsPB3:STIL^726-750^ complex for crystallography, purified STIL^726-750^ was added to purified HsPB3 in an ≳fourfold molar excess to ensure saturation. The resultant mixture was concentrated, then subjected to size exclusion in order to separate the complex from free STIL^726-750^.

### Crystallisation

STIL^726–750^ in 20 mM Tris pH 7.5, 150 mM NaCl, 2 mM TCEP was concentrated to near saturation (58.6 mg/ml as assessed by amino acid analysis). Crystals generally grew overnight, but were often overnucleated. Optimisation eventually yielded square rods with pointed tips, and single crystals, the best growing in drops containing 150 nl protein solution, 150 nl of mother liquor (7 mM HEPES pH 8.2, 93 mM Tris pH 9.0, 55.36% v/v PEG 550 MME, 10% v/v glycerol). Crystals grew overnight and were harvested after ∼1 week and flash-frozen with PEG 550 MME in the mother liquor serving as cryoprotectant.

DmPB3 in 20 mM Tris pH 7.5, 150 mM NaCl, 2 mM DTT was concentrated to 40.0 mg/ml. Crystals readily grew in many broad screen conditions, but the crystal used for structure determination grew using the Macrosol screen (Molecular Dimensions, Newmarket, UK) in a drop containing 150 nl protein solution and 50 nl mother liquor (1.5 M ammonium sulphate, 2% v/v PEG400, 100 mM Na HEPES pH 7.5). Crystals were harvested after ∼10 days and flash-frozen in liquid nitrogen using mother liquor with 30% ethylene glycol as a cryoprotectant.

apoHsPB3 in 20 mM Tris pH 7.5, 150 mM NaCl, 2 mM DTT was concentrated to 52.3 mg/ml. The best diffracting crystal example came from an optimisation screen. The drop contained 100 nl protein solution and 100 nl mother liquor [32.27% v/v PPG400 (Sigma) 100 mM NaCl 50 mM MgCl_2_]. Crystals were harvested and flash-frozen after ∼7 days with mother liquor serving as cryoprotectant.

HsPB3 in complex with STIL was purified by SEC and concentrated to 41.87 mg/ml in 20 mM Tris pH 7.5, 150 mM NaCl, 2 mM DTT. After optimisation, hexagonal rods grew in drops containing 150 nl protein solution, 50 nl mother liquor (100 mM MES pH 6.0, 191.7 mM Zn acetate, 10% v/v isopropanol). Crystals grew overnight and were harvested and flash-frozen after 3 days using mother liquor with 30% ethylene glycol as a cryoprotectant.

All experiments used the sitting drop approach at 19°C, with drops set using a Mosquito robot with a humidity chamber. Optimisation screens were based on initial hits from broad screens (Molecular Dimensions, Newmarket, UK) and were prepared using a Dragonfly robot (both robots by TTP Labtech, Melbourn, UK).

### Data collection and processing

Data were collected as described in Table 1. Datasets were integrated and scaled using the Xia2 pipeline (Winter, 2009) using XDS (Kabsch, 2010) and Aimless (Evans and Murshudov, 2013).

STIL^726-750^ was solved by molecular replacement, in Phaser (McCoy et al., 2007) using a 23-residue polyAla helix based on Ana2-CCD (PDB ID: 5AL6). This resulted in clear electron density into which a model was built using ArpWarp (Langer et al., 2008). The model was further refined in both REFMAC (Murshudov et al., 2011) and Phenix.refine (Afonine et al., 2012) with manual building in Coot (Emsley and Cowtan, 2004).

DmPB3 was solved by molecular replacement in Phaser (McCoy et al., 2007) using a CHAINSAW (Stein, 2008) model based on the polo domain from 4YYP. The model was rebuilt using BUCCANEER (Cowtan, 2006) and ArpWarp (Langer et al., 2008), and was refined in REFMAC (Murshudov et al., 2011), with manual building done in Coot (Emsley and Cowtan, 2004).

Apo HsPB3 was solved by molecular replacement. Search models were made for the HsPB3 based on either 4YYP (monomeric) or 1MBY (strand-swapped dimer). The strand-swapped dimer, but not the monomer, was able to produce convincing molecular replacement hits. The crystal had an unusually large unit cell, and using Phaser (McCoy et al., 2007) and MOLREP (Vagin and Teplyakov, 2010) it was possible to place 60 chains in the ASU, thus completing the lattice. Each chain exhibited strong non-crystallographic symmetry; however, it was not possible to average these chains by indexing to a higher crystallographic symmetry. Minor rebuilding was performed in Coot (Emsley and Cowtan, 2004) on one strand-swapped dimer before replacing this onto each chain in the ASU before further refinement, which was carried out in both REFMAC (Murshudov et al., 2011) and Phenix.refine (Afonine et al., 2012).

The HsPB3:STIL^726-750^ complex was solved by molecular replacement in Phaser (McCoy et al., 2007) using the Polo domain from 4YYP. It was possible to place three copies in the ASU. Clear helical electron density was visible for each copy, corresponding to the STIL CCD. The model was rebuilt using BUCCANEER (Cowtan, 2006) and refined in both REFMAC (Murshudov et al., 2011) and Phenix.refine (Afonine et al., 2012) with manual rebuilding performed in Coot (Emsley and Cowtan, 2004).

During refinements Molprobity (Chen et al., 2010) and PDB_REDO (Joosten et al., 2014) were used to monitor and optimise the chemical feasibility of the models.

### Analytical gel filtration

Samples of 100 µl at the indicated concentrations were injected onto an S75 10/300 column (GE Healthcare, Little Chalfont, UK) with running buffer (50 mM Tris pH 7.5, 150 mM NaCl, 2 mM DTT) flowing at 1 ml/min. Purified PB3 domains (no tags) and N-terminally His-lipoyl tagged CCD constructs were prepared at 1 mM. 30 min prior to injection, the proteins were mixed 1:1 with running buffer (apo runs), or with each other (complex runs) to give a final concentration of 500 µM in each case.

### SEC-MALS

100 µl of protein sample at the indicated concentrations was injected onto either an S200 10/300 or Superose 6 10/300 column (GE Healthcare) with running buffer (50 mM Tris pH 7.5, 150 mM NaCl, 2 mM DTT) flowing at 0.4 ml/min. The light-scattering and refractive index were respectively measured in-line by Dawn Heleos-II and Optilab rEX/TrEX instruments (Wyatt Technology, Santa Barbara, CA), as the samples eluted from the column. Data were analysed using ASTRA software (Wyatt Technology) assuming a dn/dc value of 0.186 ml/g.
